# 

**Published:** 1898-09-03

**Authors:** 


					384 THE HOSPITAL. Sept. 3> 1898.
Votloes of Sixths, Deaths, and Marriages are inserted in this
column at a charge of 2s. 6d. for an announcement not exceeding
four lines.
NOTICE TO CORRESPONDENTS.
All MB., Letters, Books for Review, and other matters intended foe
Ike Iditor should be addressed The Editoe, "The Hospital," Thx
Howital Building, 2S & 29, Southampton Stbeet, Stkand, W.O.
The Iditor cannot undertake to return refected MS., even When
accompanied by stamped directed envelope.
STotioe to Advertisers and Snbsorlbers.
All Advertisements, Orders for Copies of Thx Hospital, and Business
Communications should be addressed to TEE MAXTAQBB,
Qot to The Editor), The Hospital, The Hospital Building,28 b 29,
Southampton Street, Strand, London, W.O.
The Cost of Subscription, post free, is as followsPer week, 2Jd,
fir quarter, 2s. 8d.; per half-year, 6s. Sd.; per annum, 10s. 6d. Foreign
Per annum, 15s. 2d. s payable, in all oases, in advance.
1TOTICB.?Vol.XXIII. of " The Hospital" (October, 1897,
to March, 1898), In handsome oloth binding1, Is
now on sale at the Office of this Jonrnal, price
7s. 6d. Oases for binding1 the Numbers oontalned in
this Volnxne, Is. 6d. Index to Vol. XXIII., Sid., post
free.
The Monthly Part for September is now ready. Price
6d., by post 8d.
BOOKS RECEIVED.
ci m * i ^ . ^-HE Scientific Press.
i liysiology, Experimental and Descriptive." By Bnel P. Colton.
' . Baillieee, Tindall, and Oox.
Asepsis and Antisepsis." By H. Macnanghton Jones, M.D.
" Applied Bacteriology." By T. H. Pearmain and C. (J. Moor, M.A.
Second edition.
The "Oiiemist and DauaaiST" Offices.
" Diseases and Bemedies."
J. B. Lippincott Oo.
"System of Disease* of the Bye." Edited by William F. Norrifl,
M.D., and Oharles A. Oliver, M.D.
The Redman Publishing Tompas*.
" The Ferment Treatment of Oanoar and Taberoolosis." By Horaoe
Handera, F.K.O.8., M.D.
" Health, Loss and Gain." By M. A. Ohreiman.
Higciie and Co., Glasgow.
"Bonnie Scotland's Resorts."
400 THE HOSPITAL. Sept. 3, 1898.
The Student of Medicine.
APPOINTMENTS.
G. E. Anson, M.D., B.C.Camb., M.R.C.S.Eng.. L.R.C.P.-
Lond., appointed Honorary Surgeon to the Wellington Hospital,
New Zealand. H. Adams, L.R.C.P.Lond., appointed Honorary
Physician to the Wellington Hospital, New Zealand. Dr. J. S.
Barnes, appointed Resident Medical Officer and Public Vaccina-
tor for the Urban, Suburban, and Rural Districts of Kattanniog,
Western Australia. A. J. Beesley, L.R.C.P.Lond., M.R.C.S.Eng.,
appointed Medical Officer for the Baschurch and Middle Districts
of the Ellesmere Union. W. A. Chappie, M.B.Edin., appointed
Honorary Physician to the Wellington Hospital, New Zealand.
A. W. Connelly, M.B.Melb., appointed Resident Surgeon of
Bendigo Hospital, "Victoria. J. J. Evans, M.B.Edin., M.R.C.S.-
Eng., appointed Honorary Surgeon to the Birmingham and
Midland Eye Hospital. S. C. G. Fox, M.R.C.S., L.R.C.P.Lond.,
appointed Senior District Surgeon, Perak Federated Malay
States. E. H. Fyffe, M.B.Glasg., appointed Honorary Patholo-
gist to the Wellington Hospital, New Zealand. D. Grant, M, A.,
M.D.Edin., appointed Honorary Physician to In patients at St.
Vincent's Hospital, Melbourne. H. Hilliard, M.R.C SEng,,
L.R.C.P.Lond., appointed Assistant Anaesthetist to Charing Cross
Hospital. F. P. Kitson, L.R.C.P.Lond., M.R.C.S Eng., ap-
pointed Medical Officer for the Marshwood and Netherby Dis-
tricts of the Beaminster Union. R. G. P. Lansdown, M.D.,
B.S., M.R.C.S., L.R.C.P., appointed Lecturer on Practical
Surgery to the Medical School, University College, Bristol.
J. A. Leishman, L.R.C.P., L.R.C.S.Edin., L.F.P.S.Glasg.,
appointed Medical Officer for the Thorncombe District of the
Bedminster Union. J. H. McGee, M.D.R.U.I., D P.H.Camb.,
appointed Honorary Physician to In patients at St. Vincent's
Hospital, Melbourne. A. N. McKelvey, L.R.C P., L.R.C.S.Irel.,
appointed Assistant Medical Officer of the Omagh Lunatic
Asylum. F. A. Newman, M.B.Melb., appointed Honorary
Physician to Out-patients at St. Vincent's Hospital, Melbourne.
H. Pollen, M.D.Dub., M.Ch., L.R.C.P.I.. appointed Honorary
Physician to the Wellington Hospital, New Zealand. J. H.
Reynolds, M.B., C.M.Edin., appointed Senior House Surgeon to
the Miller Hospital, Greenwich. J. M. Schaub, M.R.C. S.Eng.,
L.R.C.P.Lond., appointed Senior Assistant Medical Officer of the
Infirmary and the Workhouse of the Parish of St. Leonard,
Shoreditch. R. N. Woodside, M.B.Melb., appointed Medical
Superintendent of the Albury Hospital, New South Wales. Dr.
A. Yelland, appointed Resident Surgeon to the Hospital,
Creswick, Victoria.
BOY All ARMY MEDICAL CORPS.
The following' are among the examination papers Bet to the candidates
for Army and Indian Medical Services at the recent examination :?
Natural Sciehces.
Zoology and Comparative Anatomy.
1. What are the chief anatomical characteristics of the following-
orders : Garnivora, Oetacea, Inseotivora, Proboscidea, Kodentia ?
2, Describe the alimentary canal and digestive process in a raminant.
S. Give a short acooant of the general strnctare of Crustacea and of
Eohinodennata.
Botany.
1. Give the characters of the following nataral orders: Ranuncula-
cece, Scrophnlariacece, Liliacece, Polygonacece, Gramineoe.
2. Describe the structure of the whole plant, including the flower and
the frnit of the common daisy (Bellis perennis), or of the wheat plant,
or of the heath plant (Erica oinerea).
8. From what sources of nutrition are the tissues of an oak tree main-
tained and increased. In what points of structure does an oak differ
from a palm tree, and from a tree fern.
Physics.
1. Describe the construction and use of voltaic cells.
Explain the following terms: electrolysis, induction, magnet,
Leyden jar.
2. Illustrate fully the conversion ot meohanical energy into heat.
S. State and explain the arguments in favour of the undulatory theory
of light.
What is the actual velooity of light ?
Geology and Physical Geography.
1. Draw up a chronological series of the tertiary beds in the British
Islands, mentioning localities where the several beds occur.
2. State the geological position and characteristic fossils of the chief
beds of true coal in the British Isles.
3. Give examples of the secular elevation of land and of the en*
croachments of the sea.

				

